# CCR2 Signal Facilitates Thymic Egress by Priming Thymocyte Responses to Sphingosine-1-Phosphate

**DOI:** 10.3389/fimmu.2018.01263

**Published:** 2018-06-07

**Authors:** Abudureyimujiang Aili, Jie Zhang, Jia Wu, Haoming Wu, Xiuyuan Sun, Qihua He, Rong Jin, Yu Zhang

**Affiliations:** ^1^Department of Immunology, School of Basic Medical Sciences, Peking University Health Science Center, Key Laboratory of Medical Immunology, Ministry of Health (Peking University), Beijing, China; ^2^Institute of Biological Sciences, Jinzhou Medical University, Jinzhou, China

**Keywords:** CCR2, Stat3, forkhead box O1, sphingosine-1-phosphate receptor 1, thymic egress

## Abstract

The signal mediated by sphingosine-1-phosphate receptor 1 (S1P1) is essential but seemingly insufficient for thymic export of newly generated T cells. Here, we reported the identification of CCR2 as an additional regulator of this process. CCR2 showed a markedly increased expression in the most mature subset of single-positive (SP) thymocytes. Its deficiency led to a reduction of recent thymic emigrants in the periphery and a simultaneous accumulation of mature SP cells in the thymus. The CCR2 signaling promoted thymic emigration primarily through modulating the chemotactic responses to S1P1 engagement. On the one hand, the chemokinesis induced by CCR2 activation endowed thymocytes with enhanced capacity to respond to S1P-induced migration. On the other hand, CCR2 signaling through Stat3 augmented forkhead box O1 activity, leading to increased expression of S1P1. Taken together, the present study highlights a unique and novel function of CCR2 signaling in the regulation of thymic egress.

## Introduction

Once seeded in the thymus, the early hematopoietic progenitors adopt a well-defined, multi-staged developmental program, which ultimately leads to the generation of a self-restricted and self-tolerant T cell repertoire of great diversity (1, 2). Smooth progression of the program is critically dependent on the signals provided by distinct thymic microenvironments mainly composed of thymic epithelial cells (TECs) (3, 4). To receive the signals at the right place and in the right order, developing thymocytes follow an intricate migratory pathway through the thymus. The chemotactic signals delivered by chemokines and other chemoattractants play an essential role in orchestrating the dynamic trafficking (5, 6).

The initial migration of early progenitors from their entry point at the cortico-medullary junction to the subcapsular region of the cortex have been suggested to be regulated by signals derived from CXCR4, CCR7, and CCR9, although none of these chemokine receptors seems to be essential for this transition (5, 6). During their outward movement, the CD4^−^CD8^−^ [double negative (DN)] progenitor cells undergo lineage specification and acquire the expression of pre-T cell receptor (pre-TCR). After the pre-TCR-mediated β-selection in the subcapsular region, cells begin to express CD4 and CD8. These double-positive (DP) thymocytes migrate back into the cortex. The reverse migration appears to be a passive process without the involvement of specific chemokine signals (5). While crawling through the cortical microenvironment, the DP thymocytes undergo positive selection, in which the nascent TCR is tested for its affinity to self-MHC presented on cortical TECs. Thymocytes bearing “useful” TCRs are rescued from apoptosis and further differentiate into either CD4^+^CD8^−^ [CD4 single-positive (CD4 SP)] or CD4^−^CD8^+^ (CD8 SP) cells (1, 2). Concomitantly, the positive selection signal induces the upregulation of CCR7. CCR7-medaited chemotaxis directs the migration of SP thymocytes into the medulla (7, 8).

In the medulla, SP thymocytes are subjected to negative selection, leading to the elimination of autoreactive T cells showing high affinity binding to self-antigens expressed by medullary TECs (3, 4). The cells that survive the negative selection undergo functional maturation to acquire immunocompetence before being exported to the periphery. The mechanisms controlling thymic emigration have been intensively studied. The chemotactic signal mediated by sphingosine-1-phosphate receptor 1 (S1P1) is an obligatory requirement for the egress of T cells from the adult thymus. Its deficiency resulted in a much reduced number of T cells in the periphery with a concomitant accumulation of mature thymocytes in the medulla (9, 10). The CCL19-activated CCR7 signal, on the other hand, mainly contributes to thymocyte emigration in newborn mice (11). The switch from CCR7- to S1P1-mediated emigration occurs approximately at 2 weeks of age during mouse ontogeny (12). What remains to be resolved is how thymic egress is appropriately controlled to ensure that only post-selected and functionally mature thymocytes are being exported. Potentially, it could be achieved by the timed expression of S1P1. At the transcriptional level, *S1pr1* is regulated by the transcription factor Kruppel-like factor 2 (KLF2), which in turn is positively regulated by the transcription factor forkhead box O1 (FoxO1) (13–16). As anticipated, deficiency in either *Klf2* or *Foxo1* causes severely impaired thymic exportation similar to that in *S1pr1*^−^*^/^*^−^ mice (10, 13, 17). However, it remains unclear of the further upstream signals that activate the FoxO1–KLF2–S1P1 axis.

Based on the expression of 6C10, CD69 and Qa-2, CD4 SP thymocytes can be resolved into four subsets, SP1 (6C10^+^CD69^+^), SP2 (6C10^−^CD69^+^), SP3 (CD69^−^Qa-2^−^), and SP4 (CD69^−^Qa-2^+^). Using multiple approaches, we have demonstrated that these four subsets each represent distinct maturation stages and together define a linear developmental pathway to be followed by the newly generated CD4 SP thymocytes (18, 19). Some previously recognized aspects have thus been unveiled, such as a potential role of *Aire* in the maturation of SP thymocytes (18). The precise dissection of the developmental pathway should also allow better understanding of such processes as thymic emigration. For example, we showed that, although *S1pr1* expression peaks in SP4 cells, significantly elevated levels of *S1pr1* mRNA is already detectable at the SP3 stage along with the upregulation of *Foxo1* and *Klf2* (19, 20). Nevertheless, thymic exportation is largely, if not exclusively, restricted to the more mature SP4 cells as evidenced by their high enrichment in recent thymic emigrants (RTEs) (12). Therefore, despite its essential role in thymic egress, S1P1 expression is not sufficient to trigger the exportation.

In search for additional signals involved in the regulation of thymic emigration, we screened the genes differentially expressed among the four subsets of CD4 SP thymocytes. CCR2 was thus identified for its restricted expression in the most mature SP4 subset. Studies with *Ccr2* knockout (KO) mice revealed an important regulatory role of CCR2 signaling in the export of mature thymocytes. This function was mediated by two independent mechanisms, both leading to enhanced cellular responses to S1P stimulation.

## Materials and Methods

### Mice

*Ccr2*-deficient mice (B6.129S4-Ccr2tm1Ifc/J, Stock No: 004999) and Rag2p-EGFP transgenic mice (FVB-Tg (Rag2-EGFP) 1Mnz/J mice, Stock No: 005688) were purchased from Jackson Laboratory (Bar Harbor, ME, USA). Rag2p-EGFP mice were backcrossed 15 generations onto the C57BL/6 background before use. B6.SJL-Ptprca PepCb (CD45.1) and C57BL/6J (CD45.2) mice were purchased from Vital River Lab Animal Technology Company (Beijing, China). Littermate female mice ranging from 4 to 13 weeks of age were used in the experiments. All mice were housed and bred in the Peking University Health Science Center animal breeding facilities (Beijing, China) under specific pathogen-free conditions. All the animal procedures were conformed to the Chinese Council on Animal Care Guidelines and the study was approved by the ethics committee of Peking University Health Science Center with an approval number of LA2017112.

### Culture Medium, Antibodies, and Reagents

RPMI-1640 medium containing 10% fetal calf serum was routinely used for primary cell cultures. KnockOut Serum Replacement (Thermo Fisher, Waltham, MA, USA) was used for all migration assays involving S1P. Anti-CD4-PE-Cy7 (GK1.5), anti-CD4-FITC (RM4–5), anti-CD8-PE (53-6.7), anti-CD8-PECF594 (53-6.7), anti-CD69 PerCP-Cy5.5 (H1.2F3), anti-CD44-PE (IM7), anti-CD62L-APC (MEL-14), and anti-p-Stat3-Percp cy5 were purchased from BD PharMingen (San Diego, CA, USA). Anti-CCR2-PE-Cy7 (SA203G11), anti-CD45.2-FITC (104), anti-CD45.1-APC (A20), anti-Qa-2-biotin (695H1-9-9), anti-Qa-2-Alexa Fluor 647 (695H1-9-9), anti-Ki67-PE-Cy7, anti-CXCR3-APC, and anti-CXCR6-PE were purchased from BioLegend (San Diego, CA, USA). Anti-CCR2-APC (Catalog # FAB5538A) and anti-S1P1-PE (Catalog # FAB7089P) were purchased from R&D Systems (Minneapolis, MN, USA). Unlabeled antibodies against Akt, p-Akt (Ser473), STAT3, p-STAT3 (Tyr705), FoxO1 (C29H4), and β-actin were purchased from Cell Signaling Technology (Beverly, MA, USA). Recombinant mouse CCL2, CXCL9, and CXCL16 were purchased from R&D Systems (Minneapolis, MN, USA). Stat3 inhibitor Stattic was purchased from Selleck.

### Flow Cytometry and Cell Sorting

Thymocytes, splenocytes, and lymph node (LN) cells were suspended in 2% newborn calf serum-balanced salt solution and stained with fluorescent Abs of various combinations. Dead cells were excluded on the basis of low forward-light scatter and propidium iodide staining. Data acquisition and analysis were performed on Gallios flow cytometer (BECKMAN COULTER) or FACS Aria II (BD). For S1P1 staining, thymocytes were stained with fluorochrome-conjugated antibodies for more than 35 min in the dark at 4°C in PBS without serum.

To enrich CD4^+^ SP thymocytes, CD8^−^ thymocytes were obtained by negative selection using anti-CD8 (Ly-2) MicroBeads (Miltenyi Biotec). The cells were then stained with fluorescently labeled antibodies to CD4, CD8, 6C10, CD69, and Qa-2. CD4 SP thymocytes (Rag2p-EGFP^+^CD4^+^CD8^−^) and its subsets SP1 (6C10^+^CD69^+^Qa-2^−^), SP2 (6C10^−^CD69^+^Qa-2^−^), SP3 (CD69^−^Qa-2^−^), and SP4 (CD69^−^Qa-2^+^) were sorted to >95% purity with a FACS Aria II (BD Biosciences, San Diego, CA, USA). SP1–3 cells used in intravital imaging were defined as CD4^+^CD8^−^Qa-2^−^.

### Quantitative PCR

Thymocyte subsets were sorted with FACS and dissolved in TRIzol (Invitrogen). The purity of RNA was verified spectrophotometrically at 260/280 nm. The RNA samples (2 µg) were reversed transcribed into cDNAs using the Reverse Transcription System (Promega). Quantitative real-time PCR was performed using iQ SYBR Green Supermix (Bio-Rad Laboratories) on an iCycler PCR system (Bio-Rad Laboratories, Hemel Hempstead, UK), with each sample in triplicate. Gapdh was used as an internal control. The following primers were used:

*Gapdh*, forward, 5′-GGAGATTGTTGCCATCAACG-3′, reverse, 5′-GATGCAGGGAT-GATGTTCTG-3′; *Ccr2*, forward, 5′-TTTGTTTTTGCAGATGATTCAA-3′, reverse, 5′-TGC-CATCATAAAGGAGCCAT-3′; *S1pr1*, forward, 5′-CAGCTCAGTCTCTGACTATG-3′, reverse, 5′-CCTTGTTGGTCAGAGTGTAG-3′; *Klf2*, forward, 5′-GCACGGATGAGGAC-CTAAAC-3′, reverse, 5′-GTAGCTGCAAGTATGTGTGG-3′; *Foxo1*, forward, 5′-GACAG-CCCTGGGTCTCAGTTT-3′, reverse, 5′-CGGGATCAACCGGTGACATA-3; *Cxcr3*, forward, 5′-TACCTTGAGGTTAGTGAACGTCA-3′, reverse, 5′-CGCTCTC-TTTTCCCCATAATC-3′; *Cxcr6*, forward, 5′-GAGTCAGCTCTGTACGATGGG-3′, reverse, 5′-TCCTTGAACTTTAGAA-CGTTT-3′; *S1pr4*, forward, 5′-GTCAGGGACTCGTACCTTCCA-3′, reverse, 5′-GATGC-ATACACACGG-3′.

### Transwell Assay

Thymocytes chemotaxis to CCL2 and S1P were performed as previously described (10). In brief, 2 × 10^5^ CD69^−^Qa-2^+^ SP4 cells were added into the top chamber of 24-well tissue culture inserts (Costar). CCL2 (100 ng/ml) or S1P (100 nM) was applied to the bottom chamber in RPMI-1640 containing 15% KnockOut Serum Replacement. After incubation at 37°C in 5% CO_2_ for 2 h, cells in the bottom chamber were collected and counted.

### Bone Marrow Reconstitution

Bone marrow cells were prepared from CD45.1 wild-type (WT) and CD45.2 Ccr2 KO mice and mixed at a ratio of 4:1. Lethally irradiated CD45.1^+^CD45.2^+^ recipient mice each received an intravenous injection of 2 × 10^6^ mixed donor cells. Eight weeks after reconstitution, mice were sacrificed and analyzed by flow cytometry.

### Intracellular Cytokine Staining

Thymocytes were first stained with fluorescently labeled antibodies against surface molecules CD4, CD8, CD69, and Qa-2 for 25 min in dark. After extensive washing, cells were fixed and permeabilized using fixation/permeabilization diluent (eBioscience, cat. no. 00-5521) according to the manufacturer’s instructions. Cells were then incubated with anti-p-Stat3-Percp cy5 (BD, San Diego, CA, USA) or anti-Ki67-PE-Cy7 (BioLegend) following the manufacturer’s recommendations.

### ELISA

Thymus was cut into pieces and milled thoroughly to get tissue homogenate. CCL2 concentration in the thymic homogenate and serum was measured using mouse CCL2 Ready Set Go ELISA Kit (eBioscience, San Diego, CA, USA) according to the manufacturer’s instructions.

### Thymic Slice Preparation and Imaging by Two-Photon Microscope

Preparation of thymic slices and imaging with a two-photon laser microscope were performed as described (21, 22). In brief, a 400-µm thymic slice was cut using a vibratome (VT1000S, LEICA), and incubated with complete medium for a few minutes at room temperature. Then the slices were placed onto a Millicell insert (30-mm organotypic PTFE; Millipore) in a 35-mm plastic Petri dish filled with 1 ml complete medium and then enclosed using silicone grease. 5 × 10^6^/ml sorted thymocytes were labeled with either 1 µM CFSE or 1 µM 5(6)-TAMRA (AAT Bioquest) in RPMI-1640 for 10 min at 37°C. The labeled thymocytes were suspended in complete medium and mixed at a ratio of 1:1, then 4 × 10^5^ mixed cells were loaded onto the thymic slice, and the cells with the slice were incubated for 2−3 h at 37°C with 5% CO_2_ to allow cells to enter the tissue.

Two-photon laser-scanning microscopy was performed with an upright TCS SP5 microscope equipped with a 20 × 1.0 NA water immersion objective (Leica) and a Chamelion VISION2 laser (COHERNT). For two-photon excitation, the Chamelion VISION2 laser was tuned to 900 nm. Imaging was performed 40–60 µm beneath the cut surface of the slice. To generate time-lapse series, samples were scanned 2.5 µm in depth 256 μm × 256 μm in width every 15–20 s for 30 min. Velocity (Perkin-Elmer, Waltham, MA, USA) was used for tracking cell positions over time in *x*–*y*–*z* three dimensions. Image stack sequences were transformed into volume-rendered four-dimensional movies. Data were calculated and analyzed using Velocity software (Perkin-Elmer, Waltham, MA, USA).

### Two-Dimensional Migration Assay

Recombinant fibronectin 50 μg/ml in PBS was used to coat pre-washed glass-bottom dishes (35 mm dish with 10 mm bottom well) at 37°C 5% CO_2_ for 2 h. The sorted and enriched WT and KO thymocytes were labeled with 1 µM 5(6)-TAMRA (AAT Bioquest) or 1 µM CFSE, suspended in 1640 medium containing 10% KO serum with or without 100 ng/ml CCL2 (R&D systems) and loaded onto pre-warmed fibronectin-coated dishes on a temperature-controlled stage. Then S1P was given with 0.3 mm capillary at one immobilized spot. Time-lapse imaging was acquired every 20 s using a confocal microscope with 488- and 562-nm lasers with Velocity software (Perkin-Elmer, Waltham, MA, USA).

### Western Blotting

Cells were lysed on ice for 30 min in the lysis buffer containing 20 mM Tris (pH 8.0), 137 mM NaCl, 5 mM Na_2_EDTA, 10% (v/v) glycerol, 1% (v/v) Triton X-100, 1 mM PMSF, 1 mM aprotinin, 1 mM leupeptin, 1 mM EGTA, 1 mM Na_3_VO_4_, 1 mM tetrasodium pyrophosphate, and 10 mM NaF. The cell lysate were resolved on SDS-PAGE and transferred to a nitrocellulose membrane. The blot was probed by incubation with primary antibodies to Akt, p-Akt (Ser473), STAT3, p-STAT3 (Tyr705), or β-Actin. HRP-conjugated anti-rabbit IgG was used as the detection antibody.

### Cell Culture and Stimulation Assay

Freshly sorted cells were washed twice with ice-cold PBS, and 2 × 10^5^ cells were seeded into 96-well plates (Costar) in 100 µl RPMI 1640 medium. CCL2 was added to 100 ng/ml. When applicable, Stat3 inhibitor Stattic was added at a concentration of 10 µM. Cells were cultured at 37°C in 5% CO_2_ for different time before analyses.

### Immunofluorescence Assay

For immunofluorescence assays, stimulated cells were fixed in 4% paraformaldehyde for 30 min at room temperature. Fixed cells were permeabilized and blocked with 0.3% Triton X-100 and 5% BSA in PBS for 1 h and incubated with anti-FoxO1 (CST) antibody at 4°C overnight. After washing, the cells were incubated with FITC-labeled goat-rabbit Ab (CST) for 1 h in dark at room temperature. Cell nuclei were stained with Hoechst33342. Then the samples were analyzed with confocal microscope.

### Data Analysis

Velocity (Perkin-Elmer) was used for three-dimensional image analysis and automated tracking of cells. The accuracy of the automated tracking was manually controlled, and only tracks with durations of >3 min were included in the analysis. Average cell velocity and displacement were calculated using Velocity. Statistical analysis was performed with GraphPad Prism 6. Results are displayed as individual data points plus median or mean ± SD or mean ± SEM. All significant values were determined using the unpaired two-tailed *t* test or calculated by ANOVA tests. Throughout the text, figures, and figure legends, the following terminology is used to denote statistical significance: *p < 0.05, **p < 0.01, ***p < 0.001; NS, no significance.

## Results

### Restricted Expression of CCR2 to Pre-Exit Mature Thymocytes

**Figure 1:**
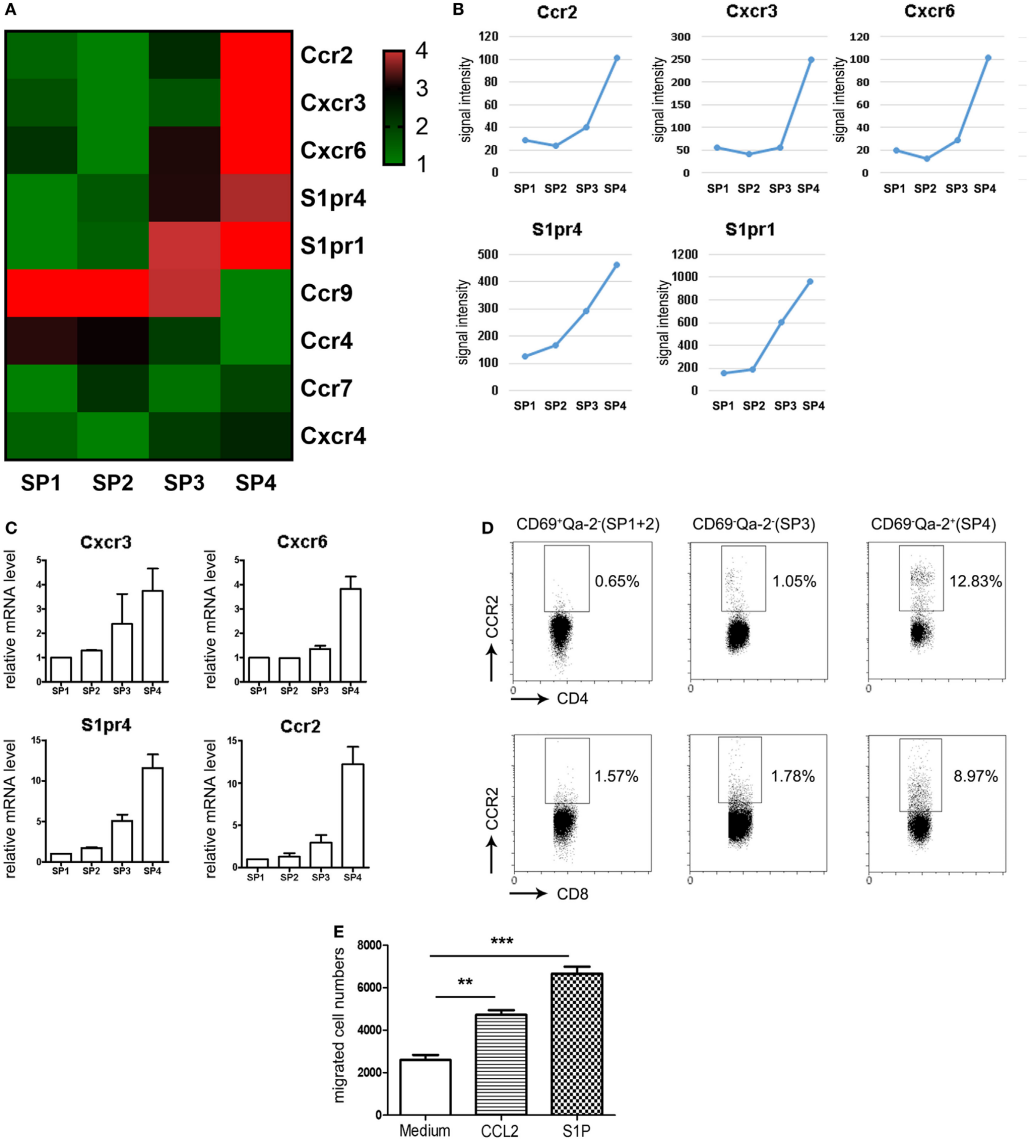
Restricted expression of CCR2 in CD69^−^Qa-2^+^ mature CD4 single-positive (SP) thymocytes. **(A,B)** Genes encoding chemoattractant receptors were sorted out from the genes differentially expressed among the four subsets (SP1–SP4) of CD4 SP thymocytes. The expression patterns of these genes are depicted in heatmap **(A)** or line charts **(B)**. **(C)** Expression levels of *Cxcr3, Cxcr6, S1pr4*, and *Ccr2* were assessed in various subsets of CD4 SP thymocytes using qPCR. The experiments were repeated three times with triplicates for each sample and the data are presented as mean ± SEM. **(D)** CCR2 expression in CD69^+^Qa-2^−^ (SP1 + SP2), CD69^−^Qa-2^−^ (SP3), and CD69^−^Qa-2^+^ (SP4) subsets of CD4 SP thymocytes as determined by flow cytometry. Representative dot plots are shown out of three independent experiments. **(E)** Purified CD69^−^Qa-2^+^ SP4 thymocytes were tested for chemotaxis to CCL2 and S1P using transwell migration assay. Data from four independent experiments with duplicates are presented as mean ± SEM. Statistical differences between groups were determined by the Student’s *t* test. **p < 0.01, ***p < 0.001.

We have previously probed the transcriptional profiles of CD4 SP thymocytes at various stages (SP1–SP4) of maturation (GEO Series accession number GSE30083) (19). In order to identify additional signals regulating thymic emigration, we revisited the microarray data by focusing on the differentially expressed genes encoding chemoattractant receptors. Since the thymic egress is largely restricted to the most mature SP4 subsets (12), special attention was given to those with elevated expression at late developmental stages. Five genes, including *Ccr2, Cxcr3, Cxcr6, S1pr1*, and *S1pr4*, were thus identified, each showing a peak expression at SP4 (Figures 1A,B). Quantitative PCR was then performed to verify their expression pattern during maturation. Similar to *S1pr1* (19), *Cxcr3* and *S1pr4* showed progressive upregulation from SP1 to SP4 (Figure 1C). *Ccr2* and *Cxcr6* mRNA, on the other hand, remained low at early stages but were sharply increased at the SP3–SP4 transition (Figure 1C). In contrast to the potent impact of S1P1 signaling in lymphocyte trafficking (9, 10), S1P4 primarily mediates the immunosuppressive effects of S1P by inhibiting T cells proliferation and secretion of effector cytokines, and no defect has been reported in cell migration in the absence of *S1pr4* (23, 24). Therefore, subsequent analyses were narrowed down on the three chemokine receptors.

To detect the surface expression of these receptors in the various subsets of SP thymocytes, flow cytometry was performed following staining with receptor-specific antibodies in combination with anti-CD4, CD8, CD69, and Qa-2. The Rag2p-EGFP mice were used here to exclude activated T cells returning to the thymus from the periphery, which is GFP^−^ in this strain (25). Consistent with their low expression at the mRNA level (not detectable until 35 cycles of PCR amplification), CXCR3^+^ or CXCR6^+^ cells were rarely seen in any subsets, including the CD69^−^Qa-2^+^ SP4 cells. On the contrary, CCR2 was readily detected in SP thymocytes following the acquisition of Qa-2 expression, with a higher positivity and more intensive staining in the CD4 lineage compared to the CD8 lineage (Figure 1D). Next, we purified CD4^+^ SP4 cells and tested their chemotactic response to CCL2, CXCL9, and CXCL16, the cognate ligands for CCR2, CXCR3, and CXCR6, respectively. Transwell migration assay confirmed that, while cells were inert to CXCL9 or CXCL16 stimulation, they were effectively mobilized by CCL2 (Figure 1E and data not shown). Together, these results identify CCR2 as a chemokine receptor primarily expressed in the most mature form of thymocytes ready to be exported to the periphery.

### Reduced Thymic Egress and Enhanced Homeostatic Expansion of Peripheral T Cells in *Ccr2*^−/−^ Mice

**Figure 2:**
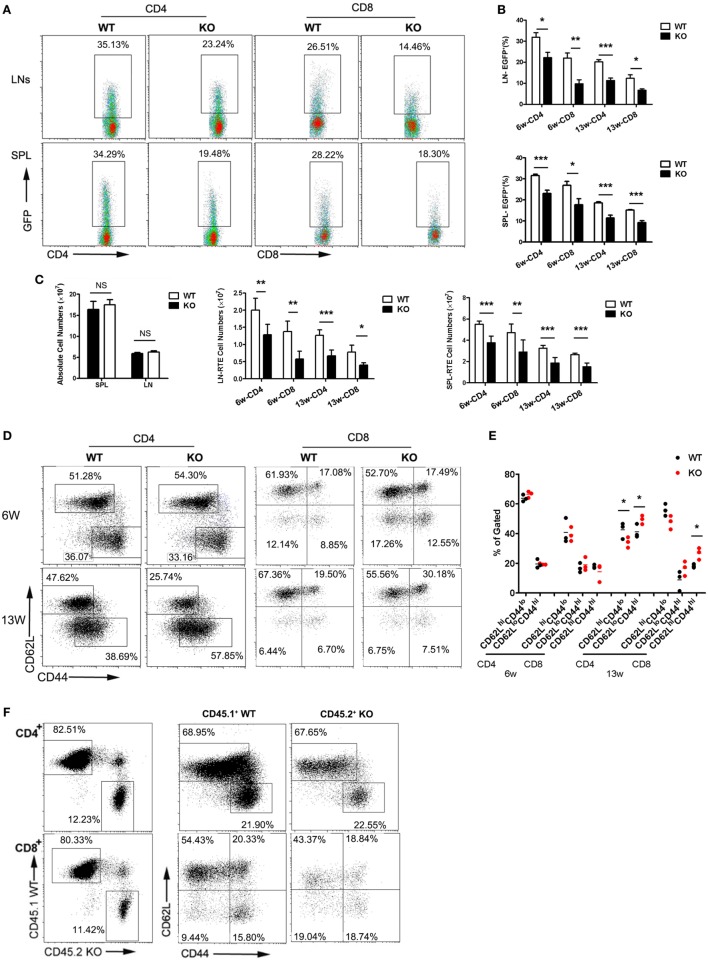
Reduced thymic output and lymphopenia-induced homeostatic expansion of peripheral T cells in *Ccr2*^−/−^ mice. Single cell suspensions were prepared from the lymph node (LN) and spleen (SPL) of wild-type (WT) and *Ccr2* knockout (KO) mice at 6 and 13 weeks of age and analyzed by flow cytometry. **(A)** Representative dot plots showing EGFP^+^ RTEs in CD4^+^ and CD8^+^ T cells from 6-week-old mice. **(B)** The percentage of RTEs in the peripheral CD4^+^ and CD8^+^ T cells in 6- and 13-week-old mice. Data are presented as mean ± SD with at least 10 mice for each group. **(C)** The absolute numbers of total and CD4^+^ and CD8^+^ RTEs in the SPL and LNs (axillary + inguinal + mesentery). **(D)** CD62L and CD44 staining for splenic CD4^+^ and CD8^+^ T cells in 6- and 13-week-old mice. The slight different gating for cells at different ages were based on the appearance of distinct populations under specific instrument settings. **(E)** Percentage of the CD62L^hi^CD44^lo^ (naive), CD62L^lo^CD44^hi^ (activated), and CD62L^hi^CD44^hi^ (memory) cells among CD4^+^ and CD8^+^ T cells. Data are presented as mean ± SEM with at least nine mice for each group. **(F)** Lethally irradiated mice were reconstituted with CD45.1^+^ WT and CD45.2^+^ KO bone marrow cells mixed at a ratio of 4:1. CD62L and CD44 expression by splenic CD4^+^ and CD8^+^ T cells were analyzed by gating on the CD45.1^+^ or CD45.2^+^ population in the recipient mice. Representative dot plots are shown out of two independent experiments with a total of nine mice. Statistical differences were determined by the Student’s *t* test. *p < 0.05, **p < 0.01, ***p < 0.001.

The restricted expression of a functional CCR2 in the SP4 subset suggests a potential role of CCR2-mediated signal in thymic emigration. To explore this possibility, we compared the RTE compartment in WT littermates and *Ccr2*-deficient mice by crossing with Rag2p-EGFP mice. Thymocytes in this strain express EGFP driven by the Rag2 promoter. Upon being exported to the periphery, they remain EGFP^+^ for 2–3 weeks, enabling easy tracking of RTEs in mice without manipulation (25). At 6 weeks of age, 24.3–37.8% (31.8% on average) of CD4^+^ cells and 15.4–26.5% (21.9% on average) of CD8^+^ cells were found to be EFGP^+^ in WT LNs. The corresponding populations dropped to 14.4–28.0% (22.2% on average) and 5.8–14.1% (9.7% on average), respectively, in the absence of *Ccr2* (Figures 2A,B). A similar reduction in EGFP positivity was also observed in the spleen (Figures 2A,B) and the liver (data not shown) of KO mice, suggesting that it was not simply the result of altered distribution of RTEs. As the mice aged, the percentage of EGFP^+^ cells decreased. The difference between WT and KO mice, however, was maintained in 13-week-old mice (Figure 2B). In line with their under-representation, the absolute number of RTE were also seen to be reduced in both LNs and spleen (Figure 2C). To rule out the possibility of a survival disadvantage associated with *Ccr2* deficiency, apoptosis was assessed with freshly isolated peripheral T cells. An equal number of Annexin V^+^ cells were present in WT and KO mice (data not shown). Therefore, the reduced number of RTEs in the KO mice is most likely the result of impaired thymocyte egress.

We further examined LN T cells for CD62L and CD44 expression. The distribution of naive (CD62L^hi^CD44^lo^), activated (CD62L^lo^CD44^hi^), and central memory (CD62L^hi^CD44^hi^) populations was comparable in WT and *Ccr2* KO mice at 6 weeks of age. However, a modest but significant increase was seen in CD4^+^ and CD8^+^ T cells with the activated/memory phenotype, with a concomitant decrease in the naive population in aged *Ccr2* KO mice (Figures 2D,E). Presumably, these phenotypic changes may either result from spontaneous activation of T cells or their homeostatic expansion under lymphopenic conditions. To distinguish these two possibilities, bone marrow chimeras were generated by reconstituting lethally irradiated mice with CD45.1^+^ WT and CD45.2^+^ *Ccr2*-deficient bone marrow cells mixed at a ratio of 4:1. Peripheral T cells in the reconstituted mice were analyzed by gating on the CD45.1^+^ or CD45.2^+^ population. As shown in Figure 2F, the skewed pattern of CD62L and CD44 staining was no longer detected with T cells derived from *Ccr2*-deficient hematopoietic progenitors in the presence of a sufficient number of WT T cells. Therefore, the distorted proportion of naive versus activated/memory T cells in *Ccr2* KO mice is primarily attributable to the homeostatic response induced by lymphopenia.

### Increased Retention of CD69^−^Qa-2^+^ Thymocytes in the Thymus of *Ccr2*^−/−^ Mice

**Figure 3:**
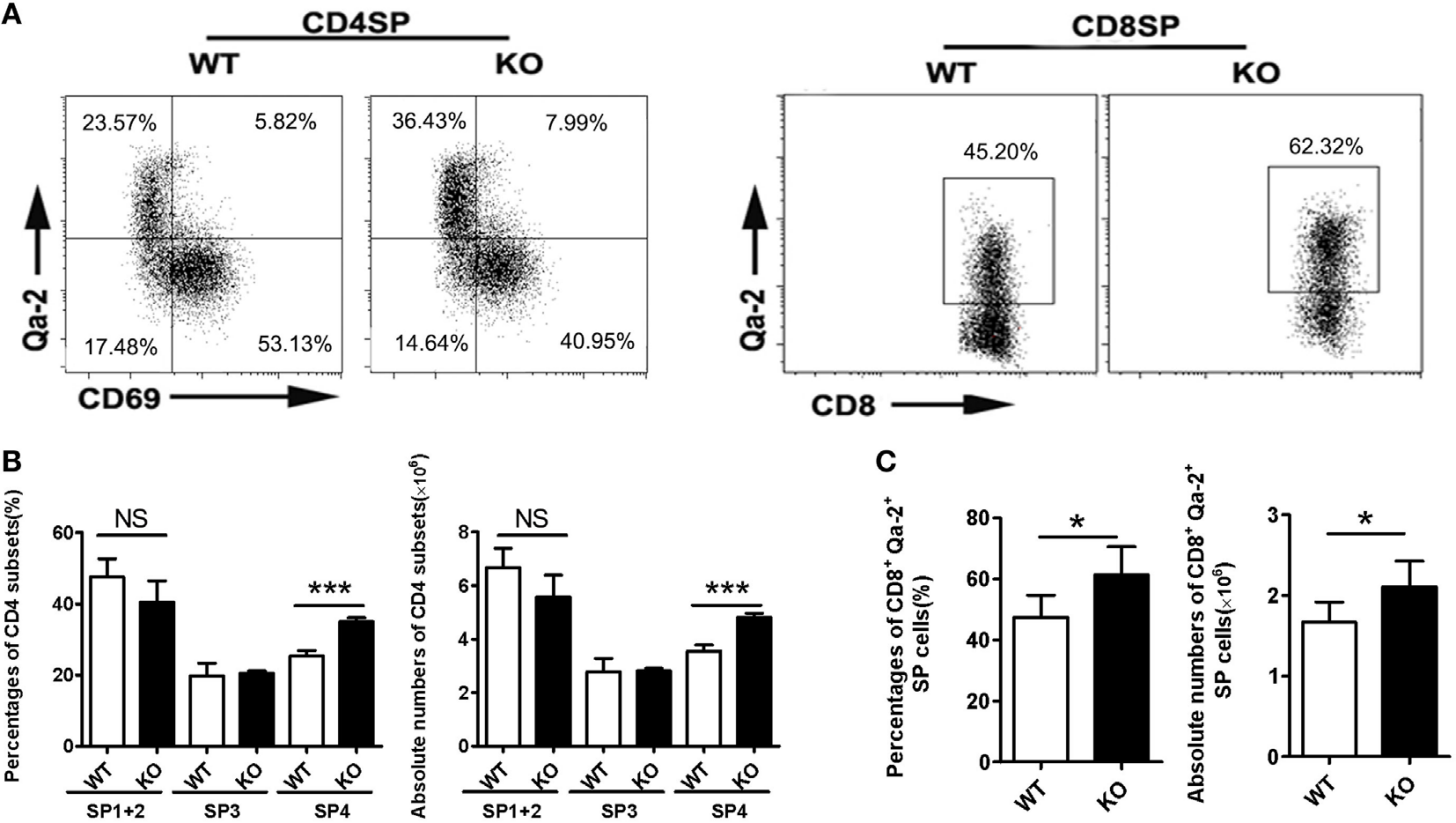
Accumulation of Qa-2^+^ mature single-positive (SP) thymocytes in *Ccr2*^−/−^ mice. **(A)** CD69 and Qa-2 staining for CD4 (*Left*) and Qa-2 staining for CD8 SP thymocytes (*Right*). **(B)** Percentages and absolute numbers of various subsets of CD4 SP cells. **(C)** Percentages and absolute numbers of CD8^+^Qa-2^+^ cells in the thymus of 6-week-old mice. Data are presented as mean ± SD with at least 10 mice for each group. *p < 0.05, ***p < 0.001.

Impaired thymic emigration is often associated with accumulation of mature thymocytes (9, 10, 13, 16). Compared to WT mice, *Ccr2* KO mice had a similar number of total thymocytes (data not shown) and were comparable in the percentages of DN, DP, CD4 SP, or CD8 SP cells (Figure S1A in Supplementary Material). When the DN population was further dissected by CD25 and CD44 staining, no difference was seen among the four subsets either (Figure S1B in Supplementary Material). On the other hand, CD69 and Qa-2 staining of the CD4 SP population did revealed a significantly elevated representation of the CD69^−^Qa-2^+^ SP4 cells, from 25.3% in the WT to 36.1% in the KO (Figures 3A,B). A similar increase was also observed for the most mature Qa-2^+^ subset in CD8 SP cells (47.5% in the WT versus 61.3% in the KO) (Figures 3A,C). Along with the increase in percentage, KO mice had higher numbers of CD4^+^CD69^−^Qa-2^+^ and CD8^+^Qa-2^+^ cells (Figures 3B,C). We next analyzed whether *Ccr2* deficiency caused any changes in cell survival and proliferation. Annexin V and Ki67 staining showed no difference in either total CD4 SP cells or the SP4 subset between WT and KO mice (Figure S2 in Supplementary Material). In addition, we examined the expression levels of several surface markers including CD3, CD28, CD45RB, CD127, and CD24 (Figure S3 in Supplementary Material), which are commonly used to monitor the maturation of SP thymocytes (25). Lack of *Ccr2* caused no overt changes in the dynamic expression of these marker except for the increased expression of Qa-2 (Figure 3A), indicating a largely normal maturation process. Taken together, these data support that the accumulation of SP4 cells most likely results from increased thymic retention.

### CCR2-Mediated Chemokinesis Promotes Directional Migration of Thymocytes in Response to S1P Stimulation

**Figure 4:**
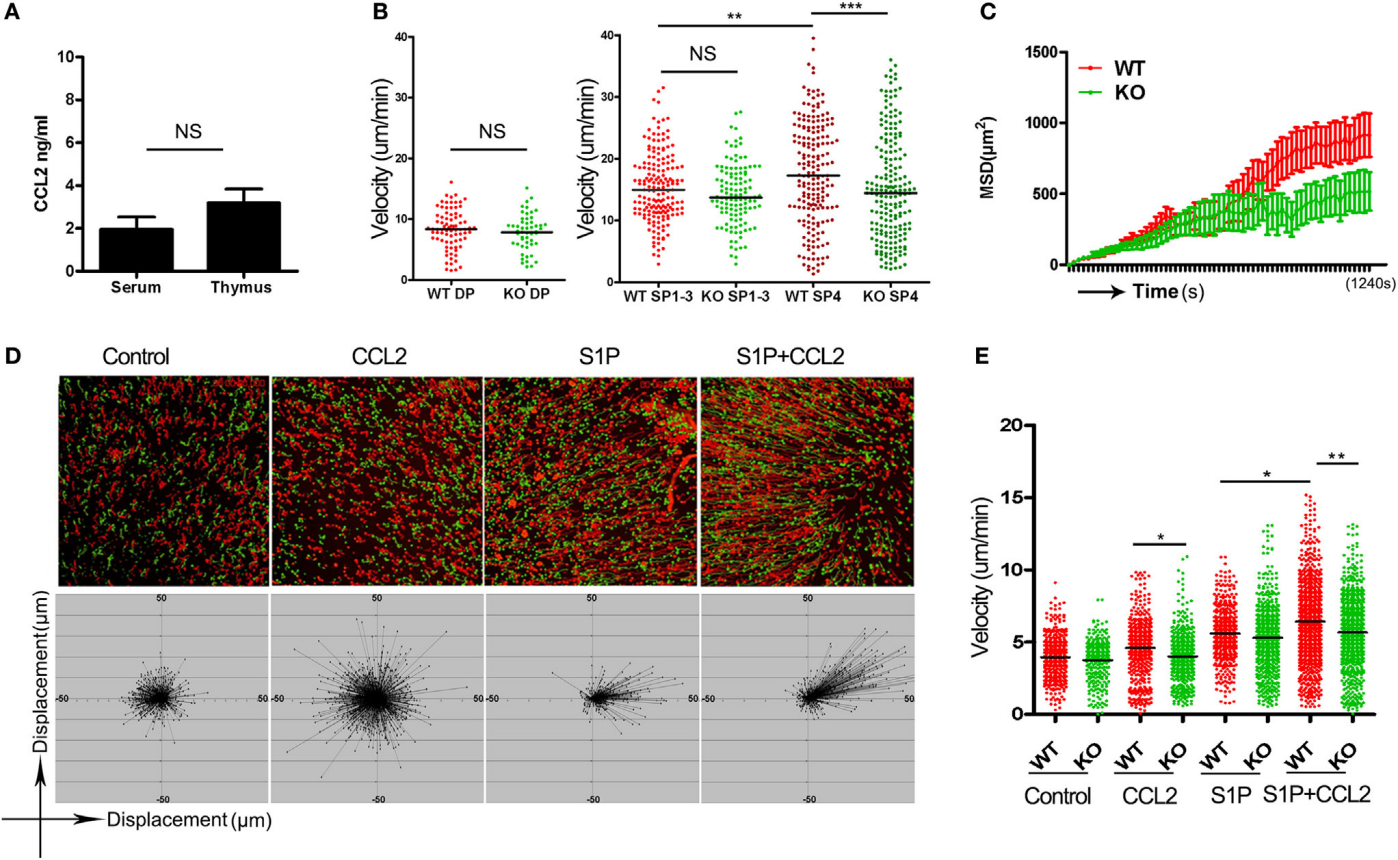
CCR2-mediated chemokinesis and its impact on S1P-induced chemotaxis. **(A)** CCL2 concentration in the thymic homogenate and serum of wild-type (WT) mice as measured by ELISA. The experiments were repeated three times with duplicates. Data are presented as mean ± SEM. **(B)** Double-positive thymocytes, and Qa-2^−^ (SP1–3) and CD69^−^Qa-2^+^ CD4 single-positive (SP) thymocytes were purified from wild-type (WT) and knockout (KO) mice and loaded onto thymic slices. Their migration speed were recorded using two-photon microscopy. The experiments were repeated three times. Each dot represents a single cell (WT SP1–3, *n* = 171; KO SP1–3, *n* = 120; WT SP4, *n* = 193; KO SP4, *n* = 198). Bars indicate the median. **(C)** Mean squared displacement (MSD) over a period of 20 min for SP4 thymocytes. Data from more than 100 cells at each time point are presented as mean ± SD. **(D)** CD69^−^Qa-2^+^ CD4 SP thymocytes were plated in a two-dimensional migration assay. Cell movement under different conditions was tracked using time-lapse imaging. The trajectories of WT (red) and KO (green) CD4 SP4 cells are shown (*Upper*). The displacement of the WT SP4 cells from the original position is depicted at the *Bottom*. **(E)** Velocity of cell movement in the two-dimensional migration assay. The experiments were repeated three times. Each dot represents a single cell. Bars indicate the median. *p < 0.05, **p < 0.01, ***p < 0.001, ns, no significance.

A straightforward explanation for the impaired thymic emigration in *Ccr2*-deficienct mice would be that CCR2 delivers a chemotactic signal critical for directional migration of the pre-exit thymocytes. Such a function would rely on the presence of a ligand gradient. The serum concentration of CCL2, the major ligand for CCR2, is estimated to be in the range of 2–4 ng/ml (26). But CCL2 is also known to be constitutively expressed by endothelial cells and TEC in the thymus (27). To ascertain whether there is a concentration gradient of CCL2 between the thymus and blood, we measured CCL2 levels in the homogenate of thymic tissues and serum using ELISA. As shown in Figure 4A, CCL2 was present at about 3 ng/ml in thymus, which was not much different from its serum concentration. This finding argues against a major role of CCR2-mediated chemotaxis in the regulation of thymic emigration.

In addition to directional migration, some chemokines may promote nondirectional movement of cells in the absence of a concentration gradient, which is referred to as chemokinesis (28). To explore the chemokinetic activity of CCL2 in the thymus, live imaging was performed with thymic slices using two-photon laser-scanning microscope (21, 22). To this end, various subsets of thymocytes were purified from WT littermates and *Ccr2* KO mice, labeled either with CFSE or 5(6)-TAMRA, and then loaded onto thymic slices prepared from CD11C-Cre^+^ ROSA26-EYFP mice, in which the medulla can be readily distinguished from the cortex on the basis of its high density of YFP^+^ cells. While the lack of *Ccr2* did not affect the cortical homing of DP cells and the medullary positioning of CD4 SP cells (data not shown), it displayed profound yet differential impacts on thymocyte motility. Consistent with the low expression of CCR2 in DP and CD4SP1–3 thymocytes, *Ccr2* deficiency caused no significant changes in the movement of their cells (Figure 4B). On the other hand, the CD69^−^Qa-2^+^ SP4 cells not only expressed high levels of CCR2 but also appeared to be the most rapidly migrating thymocyte subset with an average velocity of 17.66 µm/min. In support of an active role of CCR2 in the migration of SP4 cells, the average speed of *Ccr2*^−/−^ SP4 cells was reduced to 15.29 µm/min (Figure 4B). In addition to the lower speed, *Ccr2*^−/−^ SP4 cells showed a more confined migration (Figure 4C; Movie S1 in Supplementary Material). It should be pointed out that similar results were obtained when the dyes were swapped between experiments, ruling out the impact of different dyes on cell motility. These data indicate that the CCR2-mediated signal has a potent chemokinetic activity specifically for the most mature subset of SP thymocytes *in vivo*.

Next, we sought to elucidate how the enhanced chemokinesis might contribute to thymic egress, with a particular interest in its modulatory effect on S1P-induced migration. Fluorescence-labeled WT and *Ccr2*^−/−^ CD69^−^Qa-2^+^ SP4 cells were loaded onto a pre-warmed fibronectin-coated dish on a temperature-controlled stage and cell movement was monitored by time-lapse imaging. CCL2 was premixed with the culture medium to avoid concentration gradients. Consistent with the *in vivo* data, CCL2, in the absence of concentration gradients, significantly increased the motility of WT but not *Ccr2*^−/−^ SP4 cells (Figures 4D,E). S1P was then applied to one spot on the dish using a glass capillary. As anticipated, S1P induced directional movement of SP4 cells. Remarkably, such movement was greatly enhanced in the presence of CCL2. The enhancing effect, however, was abolished as a result of *Ccr2* deletion (Figures 4D,E; Movies S2–5 in Supplementary Material). These results support that CCR2-mediated chemokinesis endows the SP4 cells with increased responsiveness to chemotactic signals, such as S1P.

### CCR2 Signaling Promotes the Activation of the FoxO1–KLF2–S1P1 Axis Through Stat3

**Figure 5:**
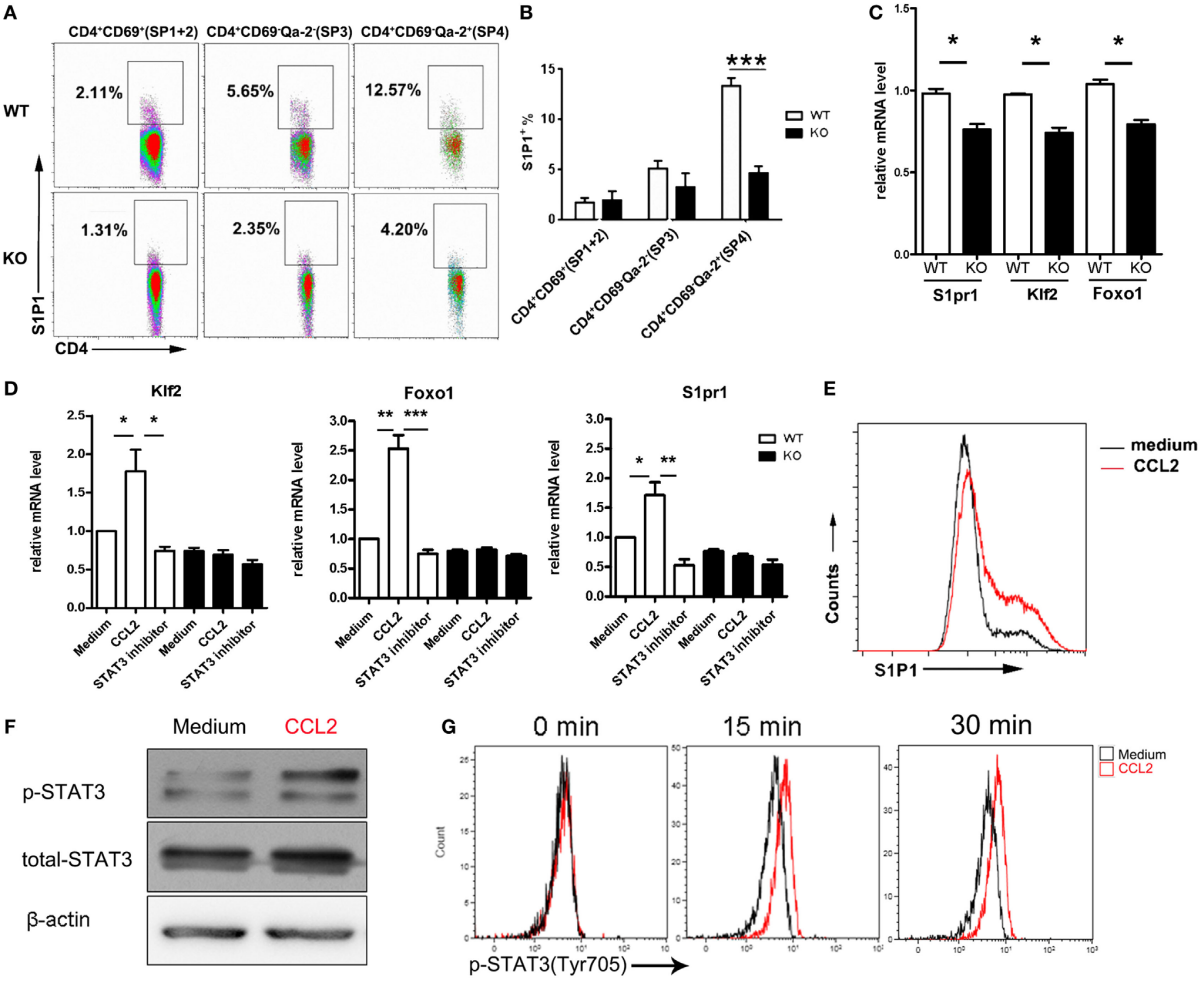

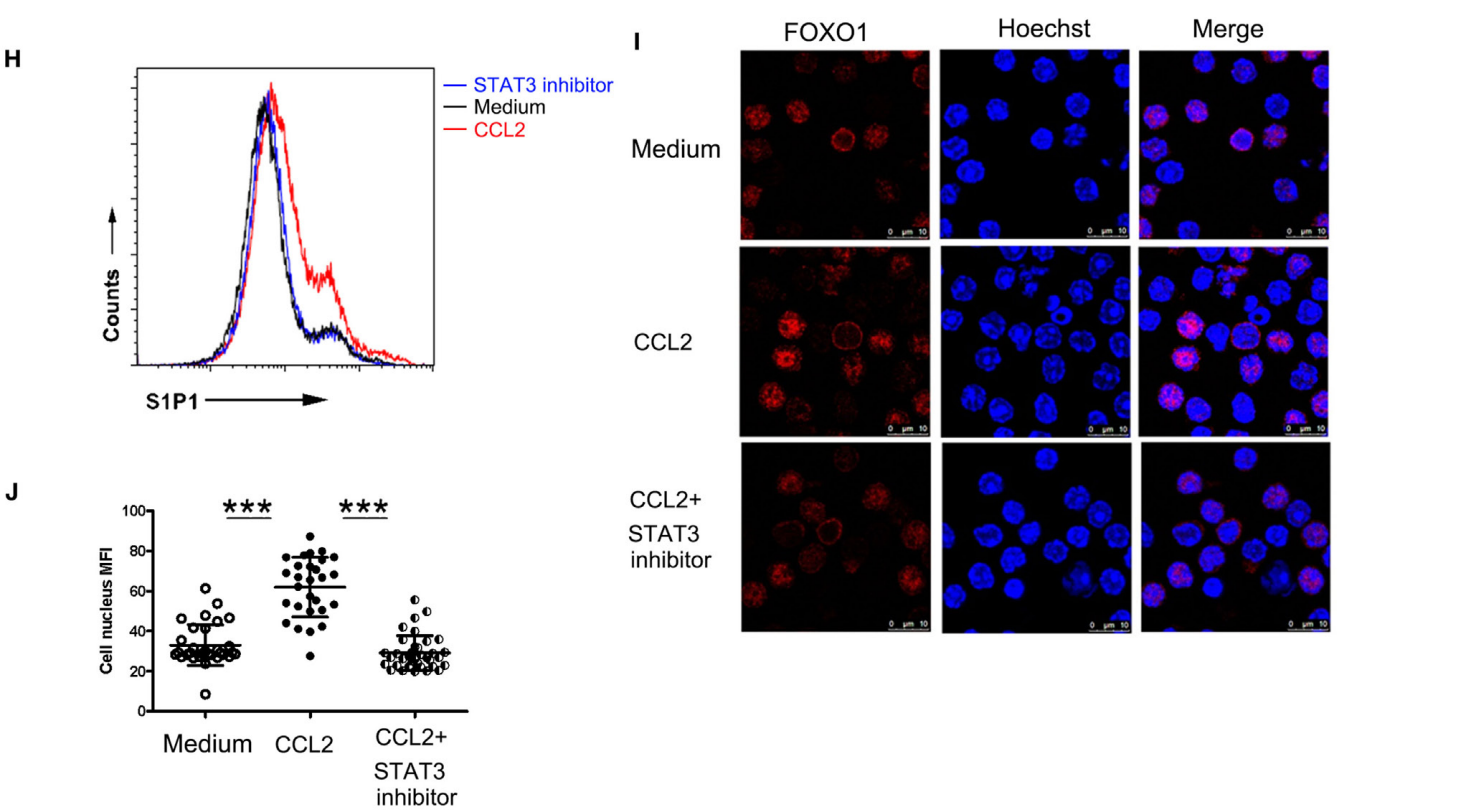
Activation of the FoxO1–KLF2–S1P1 axis by CCR2 signaling through Stat3. **(A)** Surface expression of S1P1 in the CD69^+^Qa-2^−^ (SP1 + SP2), CD69^−^Qa-2^−^ (SP3), and CD69^−^Qa-2^+^ (SP4) subsets of CD4 SP thymocytes from WT and KO mice. The experiments were repeated three times with six mice for each group. Representative dot plots are shown. **(B)** Percentage of S1P1^+^ cells are presented as mean ± SD. **(C)** Foxo1, Klf2, and S1pr1 mRNA expression in WT and KO CD4 SP thymocytes as determined by qPCR. The experiments were repeated three times with triplicates. Data are present as mean ± SEM. **(D)** CD69^−^Qa-2^+^ SP4 thymocytes from WT and KO mice were stimulated with CCL2 in the presence or absence of STAT3 inhibitor Stattic. Foxo1, Klf2, and S1pr1 mRNA expression was detected by qPCR. The experiments were repeated three times with triplicates. Data are present as mean ± SEM. **(E)** Following stimulation with CCL2, WT SP4 thymocytes were examined for surface expression of S1P1. Representative histograms are shown out of three independent experiments. **(F,G)** Levels of phosphorylated Stat3 were examined by Western blotting **(F)** or intracellular staining **(G)** in SP4 thymocytes after stimulation with CCL2. Western blotting was performed at 30 min after stimulation. The experiments were repeated three times with similar results. **(H–J)** SP4 thymocytes were stimulated with CCL2 in the presence or absence of STAT3 inhibitor Stattic. Surface expression of S1P1 was assayed by flow cytometry **(H)**. Subcellular localization of FoxO1 was revealed by immunofluorescent staining **(I)** and nucleus Foxo1 was quantified by mean fluorescent intensity (MFI) **(J)**. Data are presented as mean ± SD. Two independent experiments were performed with similar results. *p < 0.05, **p < 0.01, ***p < 0.001. Abbreviations: ns, no significance; S1P1, sphingosine-1-phosphate receptor 1; SP, single-positive; WT, wild-type; KO, knockout; KLF2, Kruppel-like factor 2; FoxO1, forkhead box O1.

It is intriguing to note that, in comparison to the WT counterparts, *Ccr2*^−/−^ SP4 cells also showed reduced migration when stimulated with S1P in the absence of CCL2, although the difference did not reach a statistical significance (Figure 4E). This raises the possibility that, in addition to inducing chemokinesis, the CCR2-mediated signal may have a direct impact on S1P signal transduction. An obvious target would be the receptor expression. Therefore, we compared the surface levels of S1P1 in CD4 SP thymocytes from *Ccr2*-deficient mice and the WT littermates. Indeed, the percentage of S1P1^+^ cells was markedly reduced in the SP3 and SP4 subsets of CD4 SP thymocytes in the absence of *Ccr2*. Moreover, the remaining S1P1^+^ cells displayed a decreased intensity of staining (Figures 5A,B). In accompany with the reduced protein expression, quantitative PCR showed the downregulation of *S1pr1* mRNA in *Ccr2*^−/−^ CD4 SP cells, although less dramatic than that at the protein level (Figure 5C). Previous studies have demonstrated that *S1pr1* transcription is activated by KLF2, which in turn is controlled by FoxO1 (14, 15). Notably, *Ccr2* deficiency also led to a decline in both *Klf2* and *Foxo1* transcripts (Figure 5B), suggesting that CCR2 signaling may represent an important upstream event in the activation of the FoxO1–KLF–S1P1 cascade.

To directly test this hypothesis, purified SP4 cells were incubated with or without the addition of CCL2. At different time points, cells were harvested and subject to PCR and FACS analyses. At an optimal concentration of 100 ng/ml, significant upregulation of *Foxo1, Klf2*, and *S1pr1* transcripts was observed as early as 30 min after CCL2 stimulation (Figure 5D). Cell surface expression of S1P1 was also found to be markedly elevated at a later time point (Figure 5E). On the other hand, their expression remained at low levels in *Ccr2*-deficient cells either in the presence or absence of CCL2 (Figure 5D), supporting the receptor specificity for its action.

As a G protein coupled receptor, CCR2 are known to exert its biological functions through multiple downstream signaling pathway, such as calcium influx, PI3K-Akt activation, and Jak–Stat activation (29–34). There is evidence that both PI3K–Akt and Jak–Stat pathways are implicated in the regulation of FoxO1 activity (35, 36). The PI3K–Akt pathway primarily serves as a negative regulator. This is best illustrated in T cells upon TCR engagement, in which phosphorylation of FoxO1 by Akt results in the nuclear exportation and subsequent degradation of FoxO1 in the cytoplasm (35). Therefore, the CCL2-induced upregulation of S1P1 is unlikely to be mediated by PI3K-Akt activation. In fact, the phosphorylated Akt remained at a basal level in SP4 thymocytes upon CCL2 stimulation when probed by Western blotting (Figure S4A in Supplementary Material).

By contrast, Stat3 is recognized as a positive regulator of the FoxO1 activity. Once activated, Stat3 directly binds to the Foxo1 promoter and activates its transcription (36). Moreover, the phosphorylated Stat3 facilitates the nuclear localization of FoxO1 (37). A previous study by Mellado et al. has demonstrated that CCL2 triggers the activation of the JAK2–STAT3 pathway in monocytes (29). Consistent with this report, enhanced phosphorylation of Stat3 was revealed in SP4 thymocytes following CCR2 engagement by Western blotting(Figure 5F) as well as intracellular staining (Figure 5G). To elucidate the role of Stat3 activation in the thymocyte response to CCL2, Stattic, a Stat3 inhibitor, was applied with CCL2. As shown in Figure 5D, the increased mRNA expression of *Foxo1, Klf2*, and *S1pr1* induced by CCL2 was abrogated with the addition of Stattic. So was the surface expression of S1P1 (Figure 5H). In addition to a potent effect on *Foxo1* transcription, CCL2 stimulation induced the accumulation of FoxO1 in the nucleus as revealed by confocal microscopy (Figures 5I,J). Of note, CCL2-induced nuclear translocation of FoxO1 was also inhibited by Stattic, confirming the involvement of Stat3. These results indicate that CCR2 primarily signals through the Jak–Stat pathway to regulate the FoxO1 activity, which ultimately leads to the increased expression of the S1P1 receptor.

## Discussion

CCR2 is known to be highly expressed in monocytes and macrophages and play a key role in their recruitment to the inflamed site (38, 39). The present study revealed a novel function of the CCR2-mediated signal in guiding thymic export of mature thymocytes. During the intramedullary maturation of SP thymocytes, CCR2 expression was found to be developmentally regulated with an abrupt increase in the most mature CD69^−^Qa-2^+^ SP4 subset. To be more accurate, only a fraction (approximately 10%) of SP4 cells was stained positive for CCR2. More intriguingly, CCR2 staining was completely overlapping with that of S1P1 (Figure S4B in Supplementary Material). In support of the functional relevance of its restricted expression, *Ccr2* deficiency was associated with a reduced RTEs compartment. As Rag2p-EGFP^+^ cells was similarly underrepresented in T cells from the LNs, spleen, and liver, this phenotype could not simply be the result of abnormal distribution of RTEs in peripheral tissues. Moreover, comparable levels of apoptotic cells were detected in RTEs from WT and KO mice, ruling out the possibility of a survival disadvantage. As such, we believe that the reduction of RTEs most likely reflects a defect in thymic egress. In accompany with a reduced RTEs compartment, the percentage, as well as the number, of CD69-Qa-2^+^ SP4 thymocytes was elevated in the absence of *Ccr2*. Since neither cell survival nor maturation was altered in such a scenario, the intrathymic accumulation of SP4 should result from their reduced export.

Thymic emigration needs to be tightly controlled so that only adequately selected and fully competent thymocytes are poised for exportation. Multiple molecules have been reported to contribute to the proper regulation of this process *via* different mechanisms. S1P1 and CCR7 deliver chemoattractatic signals to promote the exportation of adult and neonatal thymocytes, respectively (9–11). By contrast, CXCR4 contributes to the emigration of CD4 SP cells from the fetal thymus through its chemorepellent activity (40). CD69, on the other hand, inhibits thymic egress by forming a complex with S1P1 and negatively regulating S1P1 expression (41). In addition, two recent studies have reported the implication of Aire (12) and IL-4Rα (42) in thymic egress, possibly by enhancing chemokine production by thymic stromal cells. Here, we provide evidence that CCR2 signaling functions through mechanisms distinct from any of these known ones, namely by modulating thymocyte response to S1P.

First, CCR2 increases the motility of SP4 thymocytes, thereby promoting their directional movement toward S1P. Intravital imaging demonstrated that SP4 thymocytes were the most rapidly migrating cells within the thymus. Their motility, however, was significantly suppressed in the absence of CCR2, leaving them indistinguishable from SP3 thymocytes. These results suggest that CCR2 may deliver a chemokinetic signal to drive the random movement of SP4 cells. In line with this notion, CCL2, the major ligand of CCR2, strongly induces chemokinesis *in vitro* when presented homogeneously in the medium. The chemokinetic activity of CCR2 in the thymus is reminiscent of that of CCR7 in the LNs. In the latter case, the chemokinesis is proposed to contribute to rapid scanning for antigens (28, 43). While the significance of CCR2-mediated chemokinesis remains elusive, it is interesting to note that S1P-induced chemotaxis of SP4 thymocytes was markedly enhanced in the presence of CCL2. Therefore, we speculate that the increased motility induced by CCR2 may endow thymocytes with an increased responsiveness to S1P1 signaling.

Second, CCR2 mediates the upregulation of S1P1 expression. The pivotal role of S1P1 in thymic egress is manifested by the intrathymic accumulation of SP thymocytes and the peripheral T cell paucity in *S1pr1*-deficient mice. Intriguingly, similar phenotype is also reported in the absence of the transcription factor KLF2 or FoxO1 (13, 16). More in depth analyses have demonstrated that KLF2 both binds and transactivates the promoter for *S1pr1* (13, 14). KLF2, on the other hand, is a direct target of FoxO1 (15, 16). These findings suggest a regulatory axis in which FoxO1 is centrally positioned for the integration of intrinsic and environmental signals important for the control of thymic export. One of the major mechanisms for the control of FoxO1 activity involves post-translational modifications and its subcellular localization. Upon activation by phosphoinositide 3-kinase (PI3K), Akt phosphorylates FoxO1, resulting in their translocation to and sequestration in the cytoplasm (44). Taken into consideration of the fact that TCR acts as a potent activator of the PI3K–Akt pathway, Love and Bhandoola have proposed that the TCR signal induced by positively selecting interaction suppresses FoxO1 activity and the expression of KLF2 and S1P1 in semi-mature thymocytes, leading to their retention in the thymus. As TCR signaling is gradually terminated, FoxO1 activity is upregulated, thereby driving the transcription of *Klf2* and *S1pr1*. Mature SP thymocytes thus become responsive to S1P (6). In this model, the acquisition of S1P1 expression seems to be a passive and developmentally programmed process. While it is supported by the findings of impaired thymocyte export in mice with transgenic expression of constitutively active PI3K (45), the potential role of exogenous signals is not addressed in this model. As a matter of fact, several recent studies have revealed regulatory roles of cytokines in the activation of the FoxO1–KLF2–S1P1 axis. By following the differentiation from DP to CD8 SP thymocytes *in vitro*, Rafei et al. demonstrated that *Foxo1, Klf2*, and *S1pr1* transcripts were upregulated in the presence of rIL-7 and, to a lesser extent, rIL-13, whereas IL-4 had a inhibitory activity (46). However, the contribution of these cytokines remains to be determined to thymic emigration. The present study has unveiled a previously unrecognized activity of CCR2 signaling in the upregulation of S1P1. *Ccr2*-deficient SP4 cells showed a markedly reduced S1P1 expression at both mRNA and protein levels, along with a decreased expression of *Foxo1* and *Klf2* mRNA. Exogenous CCL2, on the other, enhanced their expression in a CCR2-dependent manner. In addition, more FoxO1 protein was found to be accumulated in the nucleus of CCL2-stimulation SP thymocytes. Therefore, CCL2 may affect both the transcription and nuclear translocation of FoxO1.

Various pathways are implicated in CCR2 signal transduction (29–34). Similar to what has been observed in monocytes (29), CCL2 stimulation of SP4 thymocytes induced an elevated level of phosphorylated Stat3. The implication of Stat3 in the regulation of FoxO1 activity has been suggested by several studies. In one study, Stat3 was shown to be able to bind and activate the FoxO1 promoter and its deficiency led to reduced expression of FoxO1 (36). Another study demonstrated that Stat3 is capable of interacting with FoxO1 and promoting its nuclear localization (37). Consistent with these findings, CCL2-treated SP4 thymocytes displayed an increased expression of *Foxo1* mRNA and the nuclear retention of the FoxO1 protein. More importantly, these effects, as well as the upregulation of *Klf2* and *S1pr1*, were abrogated in the presence of a Stat3 inhibitor. Stat3 activation, therefore, is required for CCL2-induced activation of the FoxO1–KLF2–S1P1 cascade. However, it should be pointed out that an early study of T cell-specific deletion of Stat3 reported no gross defect in thymocyte development (47). More detailed analysis of the SP thymocyte and RTE compartment of this mouse strain is needed to resolve the issue.

In summary, the present study has unveiled an important regulatory role of CCR2 in thymic export. First, CCR2-mediated chemokinesis enhances the chemotactic response of mature SP thymocytes to S1P stimulation. Second, CCR2 signaling through Stat3 activates the FoxO1–KLF2–S1P1 axis, leading to increased expression of S1P1. While both mechanisms facilitate thymocyte response to S1P, the CCR2-mediated chemokinesis as an acute response does not rely on the upregulation of S1P1. Identification of CCR2 as an additional regulator provides further insight into the tight control of thymic export. Future studies are warranted to understand the mechanism underlying the restricted expression of CCR2 in mature SP thymocytes.

## Ethics Statement

Littermate female mice ranging from 4 to 13 weeks of age were used in the experiments. All mice were housed and bred in the Peking University Health Science Center animal breeding facilities (Beijing, China) under specific pathogen-free conditions. All the animal procedures were conformed to the Chinese Council on Animal Care Guidelines and the study was approved by the ethics committee of Peking University Health Science Center with an approval number of LA2017112.

## Author Contributions

AA and RJ designed and carried out the study, collected and analyzed the data, and drafted the manuscript. JZ and JW performed the phenotype analysis. HW analyzed the microarray data. XS performed the cell sorting assay. QH performed the confocal and two-photon imaging. YZ conceived and designed the study, analyzed the data, and drafted the manuscript. All authors read and approved the final manuscript.

## Conflict of Interest Statement

The authors declare that the research was conducted in the absence of any commercial or financial relationships that could be construed as a potential conflict of interest.

## Supplementary Material

Figure S1 Thymocyte development in *Ccr2*-deficient mice. **(A)** Percentage of DN, DP, CD4 SP, and CD8 SP thymocytes in wild-type (WT) and knockout (KO) mice. Data are presented as mean ± SD (*n* = 12). **(B)** Percentage of DN1-4 thymocytes in WT and KO mice. Statistical data collected from 12 pairs of *Ccr2*-deficient mice and littermates were shown as mean ± SD.

Figure S2 The apoptosis and proliferation of CD4 SP thymocytes in *Ccr*2-deficient mice. Freshly isolated thymocytes were stained for CD4, CD8, CD69, Qa-2, and Annexin-V or Ki-67. The percentage of Annexin-V^+^ **(A)** and Ki-67^+^ **(B)** cells was analyzed by gating on CD4^+^CD8^−^ cells (CD4 SP) or CD4^+^CD8^−^CD69^−^Qa-2^+^ cells (CD4^+^Qa-2^+^). Representative dot plots are shown on the left. Data compiled from six pairs of mice are presented as mean ± SD on the right. NS, no significance.

Figure S3 The expression of maturation-related markers on SP thymocyte from *Ccr2* deficient mice. Thymocytes, splenocytes, and lymph node cells from 6-week-old Rag2p-EGFP *Ccr2* knockout mice (red) and the wild-type littermates (black) were analyzed by flow cytometry. Representative histogram profiles of CD4 SP **(A)** and CD8 **(B)** thymocytes are presented out of two independent experiments.

Figure S4 Akt and p-Akt levels in SP4 thymocytes. Purified SP4 thymocytes from 6-week-old C57BL/6 mice were stimulated with CCL2 or anti-CD3 Ab for 30 min. **(A)** Levels of phosphorylated Akt (p-Akt), total Akt were examined by Western blotting. The experiments were repeated three times with similar results. **(B)** The co-expression of CCR2 and S1P1 on SP4 thymocytes. Thymocytes collected from 6-week-old Rag2p-EGFP mice were stained with antibodies to CD4, CD8, CD69, Qa-2, CCR2, and S1P1. GFP^+^CD4^+^CD8^−^CD69^−^Qa-2^+^ SP4 or GFP^+^CD4^−^CD8^+^Qa-2^+^ thymocytes were gated for CCR2 and S1P1 analysis. The experiments were repeated three times with similar results.

Movie S1 Migration of CCR2^+/+^ (red) and CCR2^−/−^ (green) CD4 SP4 cells within the medulla of a live thymic slice. Time-lapse images were acquired every 20 s during 15 min detection time by two-photon microscopy. This movie correlates with Figures 4B,C.

Movie S2 Migration of CCR2^+/+^ (red) and CCR2^−/−^ (green) CD4 SP4 cells on fibronectin coated glass plates in the medium without any chemokine. Frames were acquired at 20 s intervals during 20 min detection time by confocal microscope. This movie correlates with Figures 4D,E.

Movie S3 Migration of CCR2^+/+^ (red) and CCR2^−/−^ (green) CD4 SP4 cells on fibronectin coated glass plates in the medium containing 100 ng/ml CCL2. Frames were acquired at 20 s intervals during 20 min detection time by confocal microscope. This movie correlates with Figures 4D,E.

Movie S4 Migration of CCR2^+/+^ (red) and CCR2^−/−^ (green) CD4 SP4 cells toward S1P on fibronectin coated glass plates. Frames were acquired at 20 s intervals during 20 min detection time by confocal microscope. This movie correlates with Figures 4D,E.

Movie S5 Migration of CCR2^+/+^ (red) and CCR2^−/−^ (green) CD4 SP4 cells toward S1P in the presence of homogeneously distributed CCL2 on fibronectin coated glass plates. Frames were acquired at 20 s intervals during 20 min detection time by confocal microscope. This movie correlates with Figures 4D,E.

## Abbreviations

CD4 SP, CD4^+^ single-positive thymocyte; CD8 SP, CD8^+^ single-positive thymocyte; SP1 cells, CD4^+^CD69^+^6C10^+^Qa-2^−^ cells; SP2 cells, CD4^+^CD69^+^6C10^−^Qa-2^−^ cells; SP3 cells, CD4^+^CD69^−^Qa-2^−^ cells; SP4 cells, CD4^+^CD69^−^Qa-2^+^ cells; DP, double-positive thymocyte; RTE, recent thymic emigrant.
