# Supplementary figures and images for: CCR2 Signal Facilitates Thymic Egress by Priming Thymocyte Responses to Sphingosine-1-Phosphate
Source: Front Immunol. 2018 Jun 7;9:1263. doi: 10.3389/fimmu.2018.01263 (PMC6001116; doi:10.3389/fimmu.2018.01263)

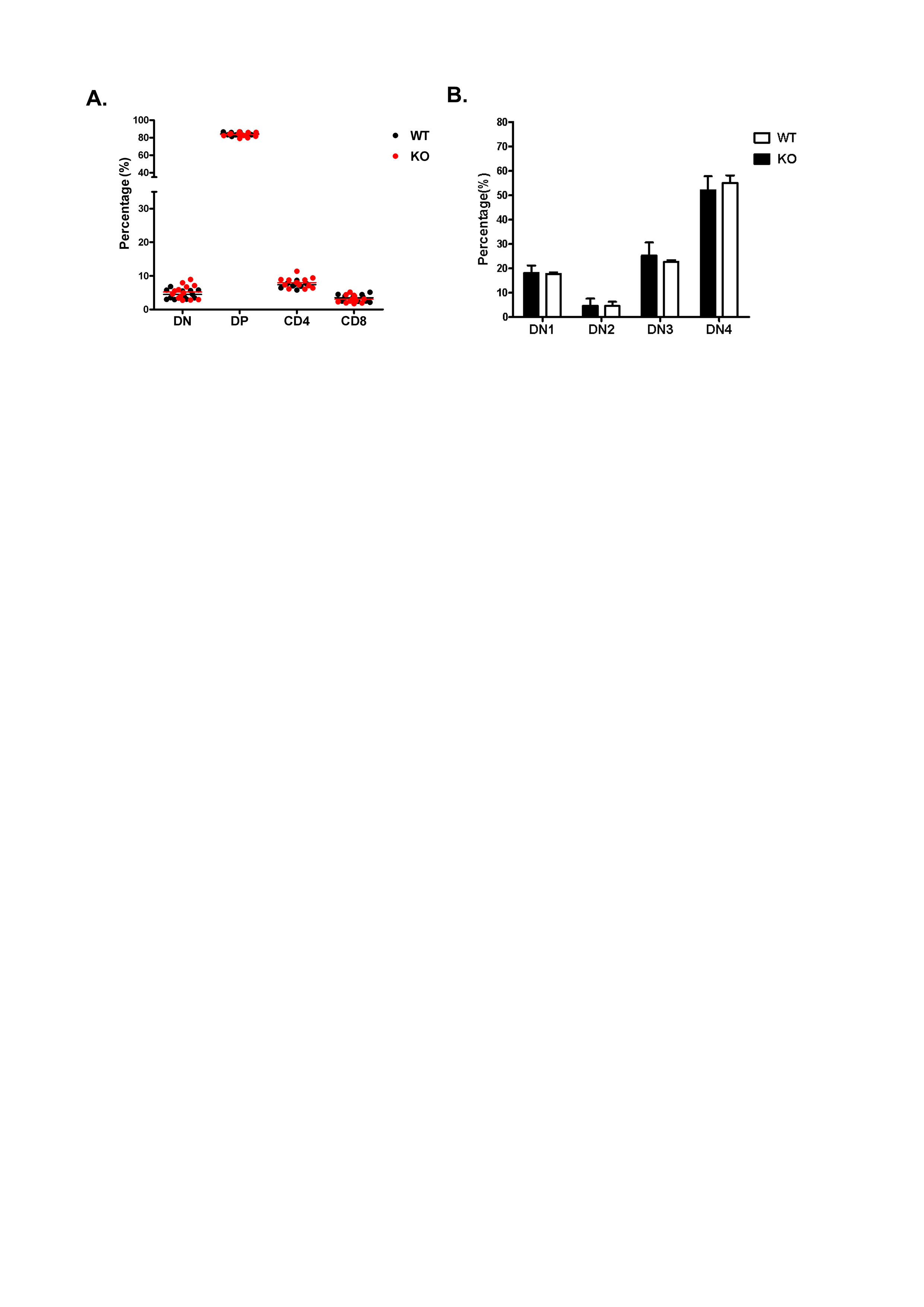

Supplement: Figure S1 — Thymocyte development in Ccr2-deficient mice. (A) Percentage of DN, DP, CD4 SP, and CD8 SP thymocytes in wild-type (WT) and knockout (KO) mice. Data are presented as mean ± SD (n = 12). (B) Percentage of DN1-4 thymocytes in WT and KO mice. Statistical data collected from 12 pairs of Ccr2-deficient mice and littermates were shown as mean ± SD. [file Image_1.TIF]

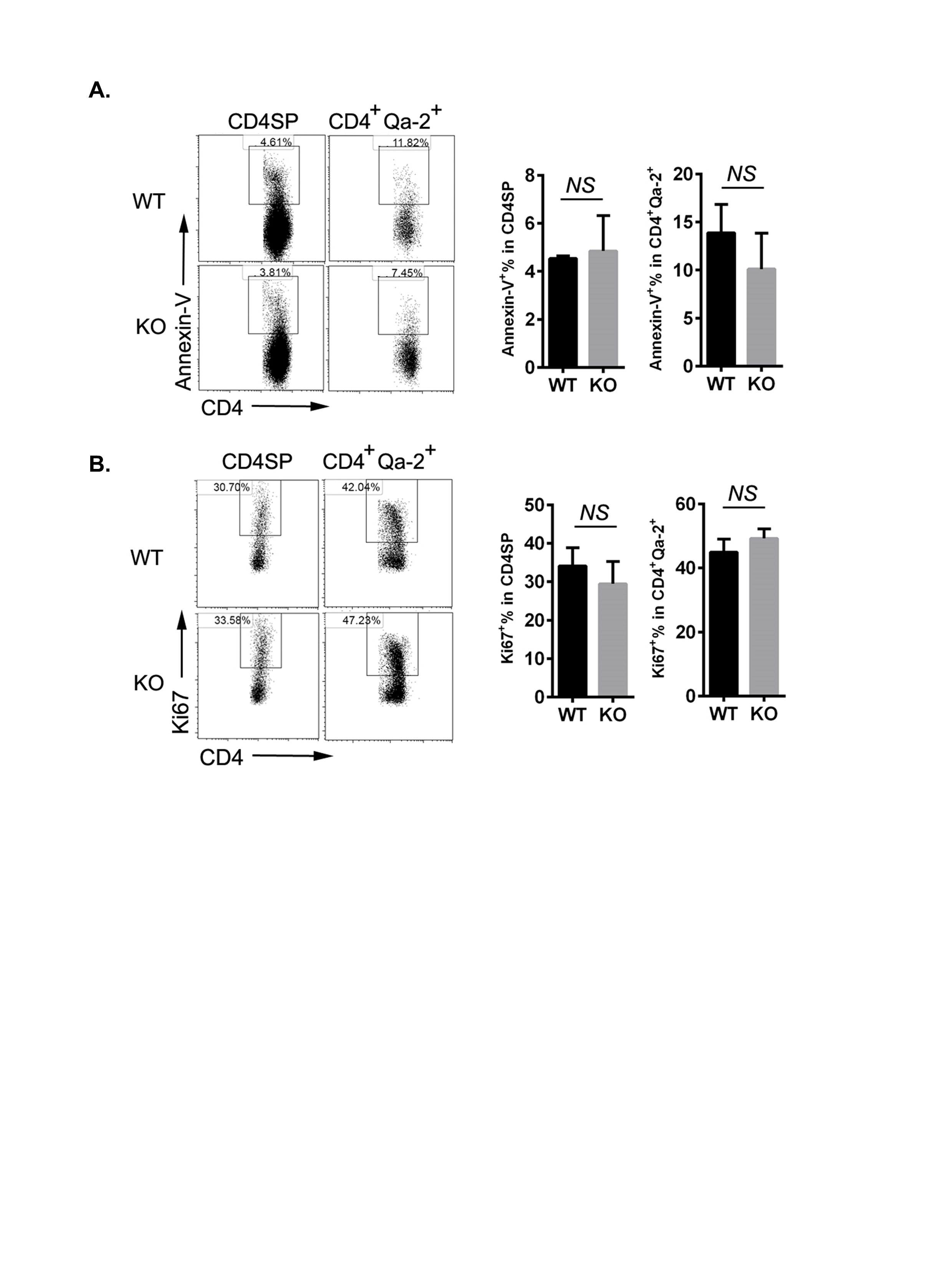

Supplement: Figure S2 — The apoptosis and proliferation of CD4 SP thymocytes in Ccr2-deficient mice. Freshly isolated thymocytes were stained for CD4, CD8, CD69, Qa-2, and Annexin-V or Ki-67. The percentage of Annexin-V+ (A) and Ki-67+ (B) cells was analyzed by gating on CD4+CD8− cells (CD4 SP) or CD4+CD8−CD69−Qa-2+ cells (CD4+Qa-2+). Representative dot plots are shown on the left. Data compiled from six pairs of mice are presented as mean ± SD on the right. NS, no significance. [file Image_2.TIF]

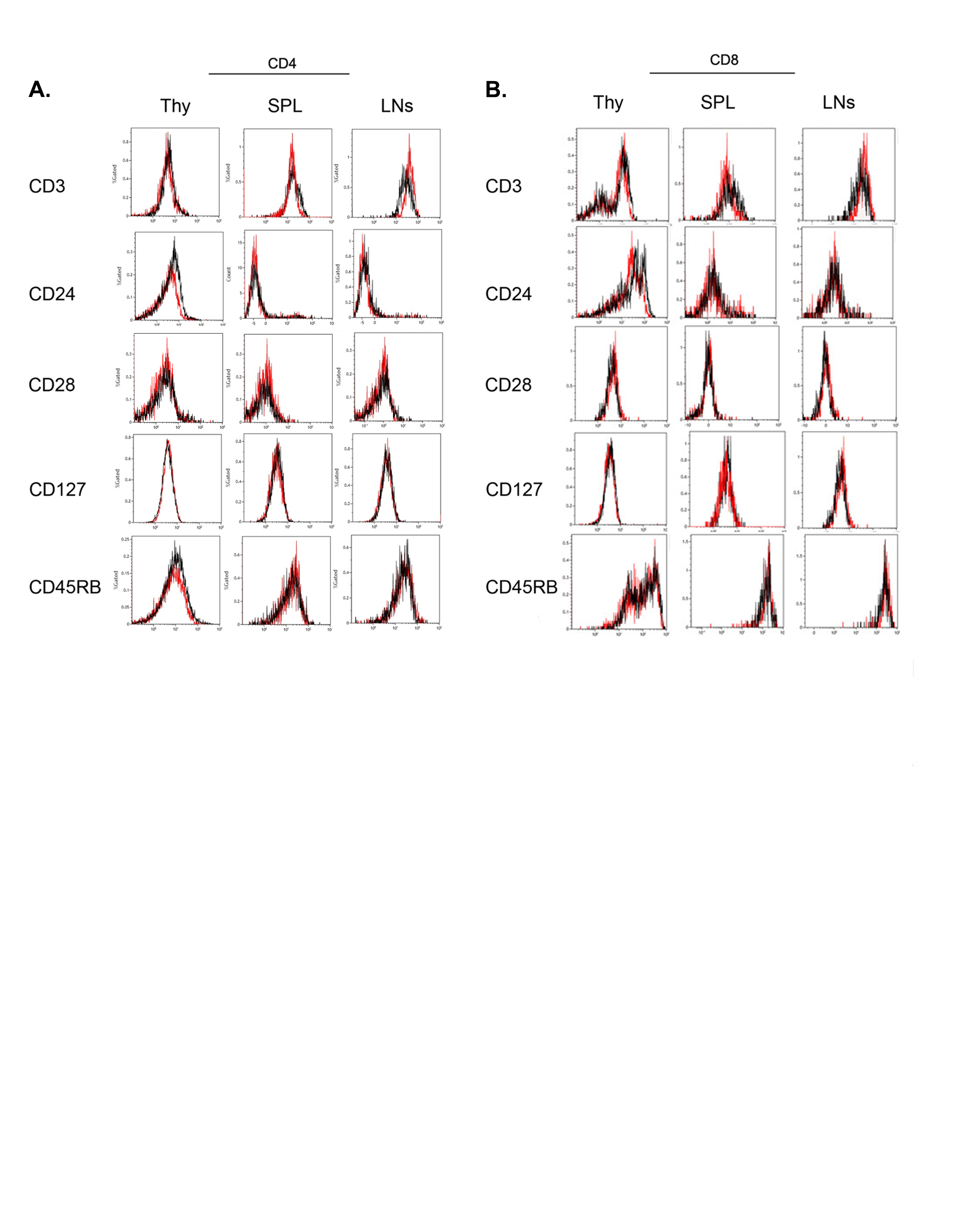

Supplement: Figure S3 — The expression of maturation-related markers on SP thymocyte from Ccr2 deficient mice. Thymocytes, splenocytes, and lymph node cells from 6-week-old Rag2p-EGFP Ccr2 knockout mice (red) and the wild-type littermates (black) were analyzed by flow cytometry. Representative histogram profiles of CD4 SP (A) and CD8 (B) thymocytes are presented out of two independent experiments. [file Image_3.TIF]

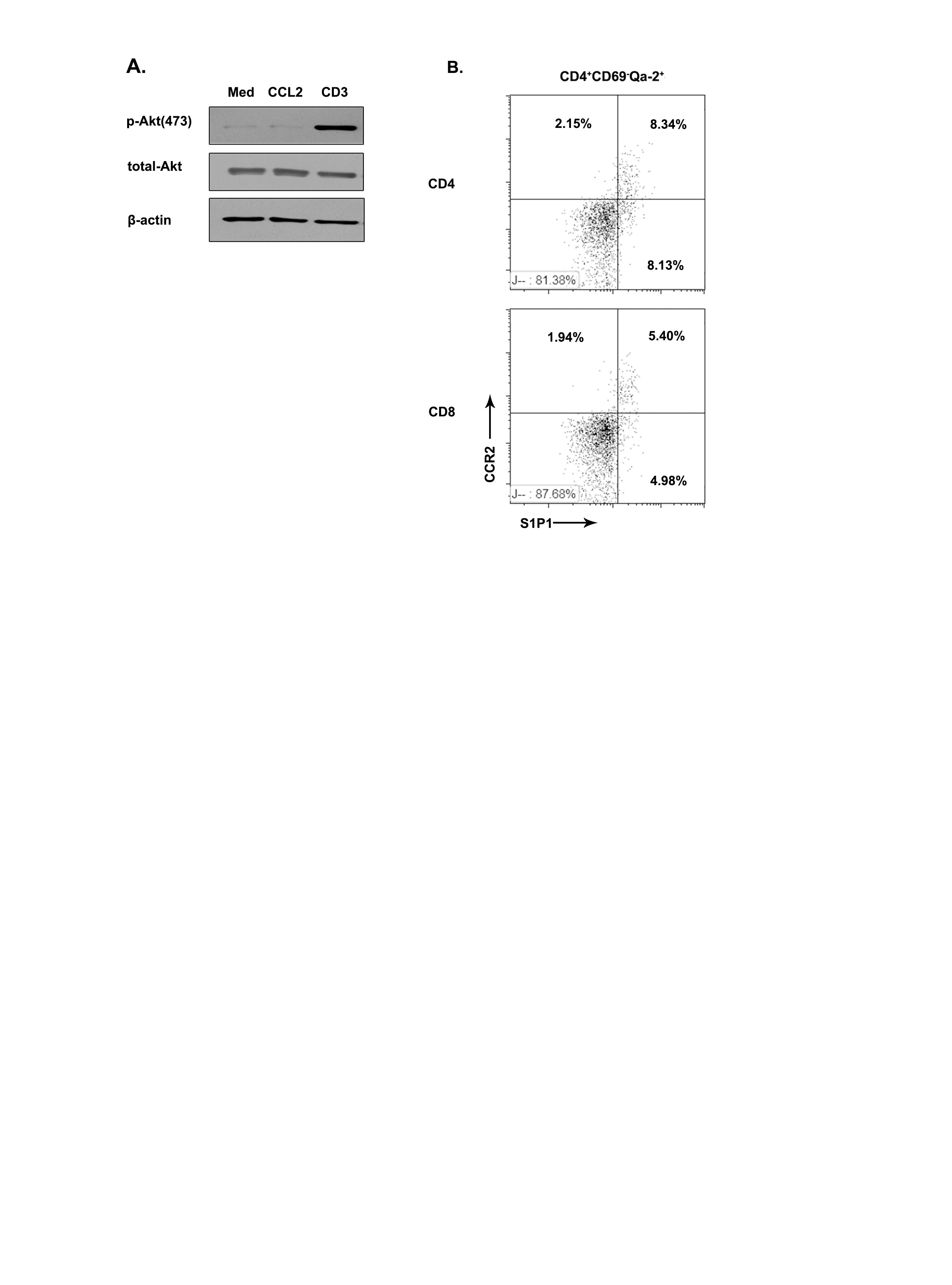

Supplement: Figure S4 — Akt and p-Akt levels in SP4 thymocytes. Purified SP4 thymocytes from 6-week-old C57BL/6 mice were stimulated with CCL2 or anti-CD3 Ab for 30 min. (A) Levels of phosphorylated Akt (p-Akt), total Akt were examined by Western blotting. The experiments were repeated three times with similar results. (B) The co-expression of CCR2 and S1P1 on SP4 thymocytes. Thymocytes collected from 6-week-old Rag2p-EGFP mice were stained with antibodies to CD4, CD8, CD69, Qa-2, CCR2, and S1P1. GFP+CD4+CD8−CD69−Qa-2+ SP4 or GFP+CD4−CD8+Qa-2+ thymocytes were gated for CCR2 and S1P1 analysis. The experiments were repeated three times with similar results. [file Image_4.TIF]
